# (3*E*)-11,16-Dioxatricyclo­[15.4.0.0^5,10^]henicosa-1(21),3,5,7,9,17,19-heptaen-2-one

**DOI:** 10.1107/S1600536813000299

**Published:** 2013-02-23

**Authors:** Gnanavelu Ganesh, Santhanagopalan Purushothaman, Piskala Subburaman Kannan, Raghavachary Raghunathan, Arunachalathevar SubbiahPandi

**Affiliations:** a Department of Physics, SMK Fomra Institute of Technology, Thaiyur, Chennai 603 103, India; b Department of Organic Chemistry, University of Madras, Guindy Campus, Chennai 600 025, India; c Department of Physics, Presidency College (Autonomous), Chennai 600 005, India

## Abstract

The title compound, C_19_H_18_O_3_, crystallizes with three mol­ecules (*A*, *B* and *C*) in the asymmetric unit. The carbonyl O atom shows positional disorder over two sites in mol­ecules *A* and *B*; the site-occupancy ratios are 0.76 (3):0.24 (3) and 0.86 (3):0.14 (3), respectively. The ethyl­ene fragments in each mol­ecule have an *E* conformation, while the C—O—C—C torsion angles indicate near planarity. The dihedral angles formed by the aromatic rings are 20.0 (1), 23.7 (1) and 16.1 (1)° for mol­ecules *A*, *B* and *C*, respectively. Intra­molecular C—H⋯O hydrogen bonds occur in each mol­ecule.

## Related literature

For the biological activities of chalcones, see: Xue *et al.* (2004 ▶); Lee *et al.* (2006 ▶); Bhat *et al.* (2005 ▶); Satyanarayana *et al.* (2004 ▶). For a related crown ether structure, see: Anh *et al.* (2011 ▶).
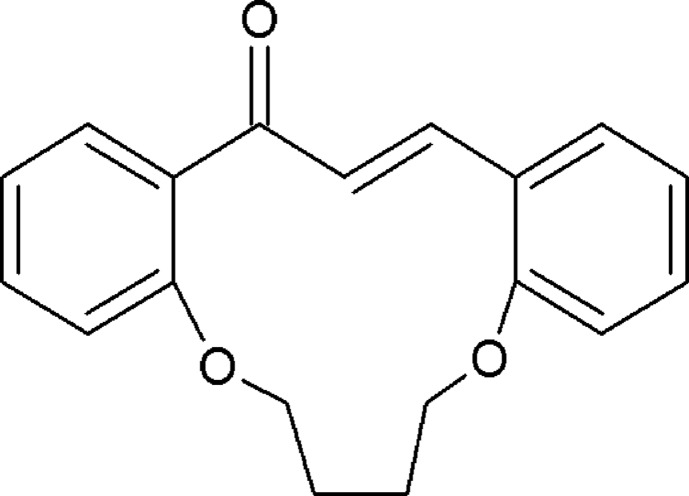


## Experimental

### 

#### Crystal data

C_19_H_18_O_3_
*M* *_r_* = 294.34
Triclinic, 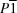

*a* = 9.0976 (6) Å
*b* = 16.9797 (11) Å
*c* = 17.2713 (9) Å
α = 62.590 (2)°
β = 88.440 (2)°
γ = 78.092 (2)°
*V* = 2310.4 (2) Å^3^
*Z* = 6
Mo *K*α radiation
μ = 0.09 mm^−1^
*T* = 293 K
0.40 × 0.30 × 0.25 mm

#### Data collection

Bruker APEXII CCD area-detector diffractometer
Absorption correction: multi-scan (*SADABS*; Bruker, 2008 ▶) *T*_min_ = 0.967, *T*_max_ = 0.979
44770 measured reflections
9919 independent reflections
5862 reflections with *I* > 2σ(*I*)
*R*_int_ = 0.032

#### Refinement

*R*[*F* ^2^ > 2σ(*F* ^2^)] = 0.045
*wR*(*F* ^2^) = 0.143
*S* = 1.04
9919 reflections
629 parameters
15 restraints
H atoms treated by a mixture of independent and constrained refinement
Δρ_max_ = 0.19 e Å^−3^
Δρ_min_ = −0.16 e Å^−3^

### 

Data collection: *APEX2* (Bruker, 2008 ▶); cell refinement: *SAINT* (Bruker, 2008 ▶); data reduction: *SAINT*; program(s) used to solve structure: *SHELXS97* (Sheldrick, 2008 ▶); program(s) used to refine structure: *SHELXL97* (Sheldrick, 2008 ▶); molecular graphics: *ORTEP-3* (Farrugia, 2012 ▶); software used to prepare material for publication: *SHELXL97* and *PLATON* (Spek, 2009 ▶).

## Supplementary Material

Crystal structure: contains datablock(s) global, I. DOI: 10.1107/S1600536813000299/bx2434sup1.cif

Structure factors: contains datablock(s) I. DOI: 10.1107/S1600536813000299/bx2434Isup2.hkl

Supplementary material file. DOI: 10.1107/S1600536813000299/bx2434Isup3.cml

Additional supplementary materials:  crystallographic information; 3D view; checkCIF report

## Figures and Tables

**Table 1:** Hydrogen-bond geometry (Å, °)

*D*—H⋯*A*	*D*—H	H⋯*A*	*D*⋯*A*	*D*—H⋯*A*
C18—H18⋯O2	0.93	2.15	2.746 (2)	121
C18—H18⋯O3	0.93	2.12	2.763 (2)	126
C38—H38⋯O5	0.93	2.24	2.753 (2)	114
C38—H38⋯O6	0.93	2.13	2.753 (3)	123
C56—H56⋯O8	0.93	2.23	2.766 (2)	116
C56—H56⋯O9	0.93	2.15	2.764 (2)	122

**Fig. 1.**
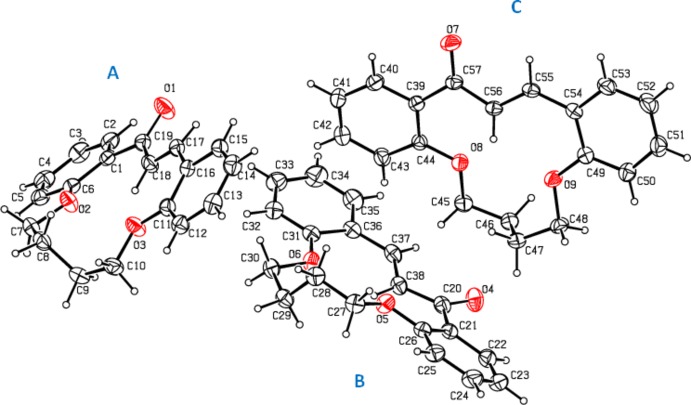
The structure of the title compound, showing the atom-numbering scheme. The displacement ellipsoids are drawn at the 30% probability level.

## supplementary crystallographic information

### Comment

Chalcone derivatives possess a wide range of biological properties such as antimalarial (Xue *et al.*, 2004), antiangiogenic and antitumour (Lee *et al.*, 2006), anticancer (Bhat *et al.*, 2005) and antihyperglycemic (Satyanarayana *et al.*, 2004) activities. Chalcones have been widely studied and developed as one of the pharmaceutically important molecules. Herein we report the crystal structure of the title compound.

The macomolecular structure of the title compound, C_57_H_52_O_9_, includes 14-crown-2-ether skeletal moiety (Fig. 1). The bond lengths and bond angles are comparable with the related crown ether structure (Anh *et al.*, 2011). The compound crystallizes with three independent molecules (A, B and C) per asymmetric unit. Out of three molecules, two molecules are disordered with two alternate positions for the O atoms [atom O1/O1' in molecule A, and O4/O4' in molecule B] with site occupancies are 0.76/0.24 and 0.86/0.14. The ethylene fragments in all the three molecules have E configuration, while the C—O—C—C torsion angles indicate planarity of the segments. The dihedral angles between the aromatic rings are 20.0 (1), 23.7 (1) and 16.1 (1)° for molecules A, B and C, respectively.

Each molecule of the asymmetric unit exhibits intramolecular C—H···O hydrogen bonds [Table 1].

### Experimental

To a solution of 2 g (5.08 mmol) of 2-(4-(4-((2-acetylphenoxy) methyl)-1*H*-1,2,3-triazol-1-yl) butoxy)benzaldehyde in 30 ml of methanol, 0.30 g (7.63 mmol) of NaOH in 30 ml of methanol was added drop wise and the mixture was stirred vigorously at room temperature for about 8 h. After the completion of the reaction, as evidenced by TLC analysis, the solvent was removed under vacuum. The crude product was then subjected to column chromatography using petroleum ether/ethylacetate (1:1) as eluent. The single crystal suitable for X-ray diffraction were obtained by slow evaporation of a solution of the title compound in methanol at room temperature.

### Refinement

All H atoms were fixed geometrically and allowed to ride on their parent C atoms, with C—H distances fixed in the range 0.93–0.97 Å with *U*_iso_(H) = 1.2*U*_eq_(C) for other H atoms. The carbonyl group O atoms of two molecules shows positional disorder and were refined split into two positions with occupancies of 0.76 (3)/0.24 (3) and 0.86 (3)/0.14 (3).

### Crystal data

**Table:**

C_19_H_18_O_3_	*Z* = 6
*M**_r_* = 294.34	*F*(000) = 936
Triclinic, *P*1	*D*_x_ = 1.269 Mg m^−^^3^
Hall symbol: -P 1	Mo *K*α radiation, λ = 0.71073 Å
*a* = 9.0976 (6) Å	Cell parameters from 9919 reflections
*b* = 16.9797 (11) Å	θ = 2.0–26.9°
*c* = 17.2713 (9) Å	µ = 0.09 mm^−^^1^
α = 62.590 (2)°	*T* = 293 K
β = 88.440 (2)°	Block, white crystalline
γ = 78.092 (2)°	0.40 × 0.30 × 0.25 mm
*V* = 2310.4 (2) Å^3^	

### Data collection

**Table:**

Bruker APEXII CCD area-detector diffractometer	9919 independent reflections
Radiation source: fine-focus sealed tube	5862 reflections with *I* > 2σ(*I*)
Graphite monochromator	*R*_int_ = 0.032
ω and φ scans	θ_max_ = 26.9°, θ_min_ = 1.4°
Absorption correction: multi-scan (*SADABS*; Bruker, 2008)	*h* = −11→11
*T*_min_ = 0.967, *T*_max_ = 0.979	*k* = −21→21
44770 measured reflections	*l* = −21→21

### Refinement

**Table:**

Refinement on *F*^2^	Primary atom site location: structure-invariant direct methods
Least-squares matrix: full	Secondary atom site location: difference Fourier map
*R*[*F*^2^ > 2σ(*F*^2^)] = 0.045	Hydrogen site location: inferred from neighbouring sites
*wR*(*F*^2^) = 0.143	H atoms treated by a mixture of independent and constrained refinement
*S* = 1.04	*w* = 1/[σ^2^(*F*_o_^2^) + (0.063*P*)^2^ + 0.2901*P*] where *P* = (*F*_o_^2^ + 2*F*_c_^2^)/3
9919 reflections	(Δ/σ)_max_ < 0.001
629 parameters	Δρ_max_ = 0.19 e Å^−^^3^
15 restraints	Δρ_min_ = −0.16 e Å^−^^3^

### Special details

**Table:**

Geometry. All esds (except the esd in the dihedral angle between two l.s. planes) are estimated using the full covariance matrix. The cell esds are taken into account individually in the estimation of esds in distances, angles and torsion angles; correlations between esds in cell parameters are only used when they are defined by crystal symmetry. An approximate (isotropic) treatment of cell esds is used for estimating esds involving l.s. planes.
Refinement. Refinement of F^2^ against ALL reflections. The weighted R-factor wR and goodness of fit S are based on F^2^, conventional R-factors R are based on F, with F set to zero for negative F^2^. The threshold expression of F^2^ > 2sigma(F^2^) is used only for calculating R-factors(gt) etc. and is not relevant to the choice of reflections for refinement. R-factors based on F^2^ are statistically about twice as large as those based on F, and R- factors based on ALL data will be even larger.

### Fractional atomic coordinates and isotropic or equivalent isotropic displacement parameters (Å^2^)

**Table:**

	*x*	*y*	*z*	*U*_iso_*/*U*_eq_	Occ. (<1)
C1	0.2486 (2)	0.55742 (11)	0.08728 (12)	0.0588 (5)	
C2	0.3295 (3)	0.56983 (13)	0.01475 (14)	0.0739 (6)	
H2	0.4339	0.5592	0.0212	0.089*	
C3	0.2613 (3)	0.59712 (15)	−0.06609 (15)	0.0862 (7)	
H3	0.3189	0.6037	−0.1132	0.103*	
C4	0.1071 (3)	0.61454 (15)	−0.07661 (15)	0.0819 (6)	
H4	0.0599	0.6341	−0.1314	0.098*	
C5	0.0222 (3)	0.60341 (13)	−0.00704 (13)	0.0705 (5)	
H5	−0.0822	0.6151	−0.0149	0.085*	
C6	0.0908 (2)	0.57491 (12)	0.07496 (12)	0.0604 (5)	
C7	−0.1490 (2)	0.58850 (16)	0.13795 (14)	0.0746 (6)	
H7A	−0.1910	0.5576	0.1115	0.089*	
H7B	−0.1840	0.6536	0.1023	0.089*	
C8	−0.1943 (2)	0.55946 (15)	0.22954 (14)	0.0751 (6)	
H8A	−0.3007	0.5853	0.2272	0.090*	
H8B	−0.1389	0.5840	0.2573	0.090*	
C9	−0.1666 (2)	0.45690 (16)	0.28566 (15)	0.0799 (6)	
H9A	−0.2619	0.4386	0.2900	0.096*	
H9B	−0.1012	0.4281	0.2563	0.096*	
C10	−0.0971 (2)	0.42235 (16)	0.37628 (14)	0.0788 (6)	
H10A	−0.1556	0.4536	0.4057	0.095*	
H10B	−0.0919	0.3577	0.4105	0.095*	
C11	0.1433 (2)	0.42178 (13)	0.43431 (13)	0.0630 (5)	
C12	0.1029 (2)	0.38431 (14)	0.51991 (13)	0.0713 (5)	
H12	0.0078	0.3714	0.5316	0.086*	
C13	0.2019 (3)	0.36627 (15)	0.58727 (14)	0.0776 (6)	
H13	0.1743	0.3402	0.6445	0.093*	
C14	0.3413 (3)	0.38636 (16)	0.57095 (15)	0.0823 (6)	
H14	0.4080	0.3749	0.6167	0.099*	
C15	0.3817 (2)	0.42369 (13)	0.48619 (14)	0.0720 (5)	
H15	0.4765	0.4372	0.4757	0.086*	
C16	0.2861 (2)	0.44200 (12)	0.41563 (12)	0.0601 (5)	
C17	0.3411 (2)	0.47976 (13)	0.32785 (14)	0.0654 (5)	
C18	0.2732 (2)	0.49489 (13)	0.25304 (13)	0.0716 (6)	
H18	0.1781	0.4823	0.2549	0.086*	
C19	0.3368 (2)	0.52898 (12)	0.17062 (13)	0.0663 (5)	
C20	0.7253 (2)	−0.13378 (14)	0.66919 (13)	0.0646 (5)	
C21	0.6521 (2)	−0.17445 (13)	0.75325 (13)	0.0626 (5)	
C22	0.6970 (3)	−0.26840 (16)	0.80557 (16)	0.0811 (6)	
H22	0.7659	−0.3022	0.7855	0.097*	
C23	0.6422 (3)	−0.31261 (17)	0.88618 (18)	0.0928 (7)	
H23	0.6719	−0.3754	0.9190	0.111*	
C24	0.5441 (3)	−0.26358 (19)	0.91737 (18)	0.0963 (7)	
H24	0.5100	−0.2930	0.9726	0.116*	
C25	0.4957 (3)	−0.17205 (16)	0.86847 (15)	0.0809 (6)	
H25	0.4280	−0.1395	0.8903	0.097*	
C26	0.5470 (2)	−0.12669 (14)	0.78589 (13)	0.0644 (5)	
C27	0.3856 (2)	0.01519 (14)	0.76303 (13)	0.0705 (5)	
H27A	0.4206	0.0144	0.8160	0.085*	
H27B	0.2971	−0.0108	0.7746	0.085*	
C28	0.3489 (2)	0.11039 (14)	0.69007 (12)	0.0674 (5)	
H28A	0.4423	0.1304	0.6721	0.081*	
H28B	0.2909	0.1495	0.7121	0.081*	
C29	0.2608 (2)	0.12287 (15)	0.60977 (13)	0.0698 (5)	
H29A	0.2617	0.0635	0.6148	0.084*	
H29B	0.1569	0.1520	0.6090	0.084*	
C30	0.3217 (2)	0.17845 (14)	0.52471 (12)	0.0666 (5)	
H30A	0.2539	0.1920	0.4757	0.080*	
H30B	0.3349	0.2352	0.5213	0.080*	
C31	0.5525 (2)	0.15677 (13)	0.45736 (12)	0.0564 (4)	
C32	0.5141 (2)	0.24309 (14)	0.38767 (12)	0.0672 (5)	
H32	0.4224	0.2812	0.3844	0.081*	
C33	0.6110 (3)	0.27270 (17)	0.32327 (15)	0.0850 (6)	
H33	0.5843	0.3307	0.2763	0.102*	
C34	0.7463 (3)	0.2176 (2)	0.32775 (18)	0.1040 (8)	
H34	0.8125	0.2382	0.2845	0.125*	
C35	0.7843 (3)	0.13101 (18)	0.39687 (16)	0.0888 (7)	
H35	0.8769	0.0940	0.3994	0.107*	
C36	0.6886 (2)	0.09715 (14)	0.46310 (12)	0.0611 (5)	
C37	0.7366 (2)	0.00422 (16)	0.53391 (14)	0.0654 (5)	
C38	0.6604 (2)	−0.04329 (13)	0.60039 (12)	0.0615 (5)	
H38	0.5610	−0.0171	0.6027	0.074*	
C39	0.93750 (19)	0.19043 (12)	0.74456 (11)	0.0533 (4)	
C40	0.8673 (2)	0.28237 (13)	0.69700 (13)	0.0678 (5)	
H40	0.8908	0.3236	0.7134	0.081*	
C41	0.7645 (2)	0.31371 (15)	0.62675 (15)	0.0797 (6)	
H41	0.7184	0.3751	0.5969	0.096*	
C42	0.7305 (2)	0.25418 (16)	0.60102 (15)	0.0810 (6)	
H42	0.6623	0.2754	0.5529	0.097*	
C43	0.7958 (2)	0.16383 (14)	0.64527 (13)	0.0697 (5)	
H43	0.7717	0.1240	0.6270	0.084*	
C44	0.89803 (19)	0.13060 (12)	0.71742 (11)	0.0546 (4)	
C45	0.9246 (2)	−0.02235 (13)	0.73831 (13)	0.0680 (5)	
H45A	0.9565	−0.0116	0.6809	0.082*	
H45B	0.8162	−0.0159	0.7369	0.082*	
C46	1.0010 (2)	−0.11563 (13)	0.80595 (13)	0.0671 (5)	
H46A	1.1071	−0.1172	0.8130	0.081*	
H46B	0.9933	−0.1587	0.7848	0.081*	
C47	0.9366 (2)	−0.14613 (13)	0.89507 (13)	0.0699 (5)	
H47A	0.8698	−0.0941	0.8960	0.084*	
H47B	0.8769	−0.1901	0.9030	0.084*	
C48	1.0545 (2)	−0.18769 (13)	0.97021 (13)	0.0692 (5)	
H48A	1.0096	−0.2139	1.0256	0.083*	
H48B	1.1307	−0.2349	0.9666	0.083*	
C49	1.2255 (2)	−0.12862 (13)	1.02307 (11)	0.0564 (4)	
C50	1.2922 (2)	−0.21385 (14)	1.08800 (13)	0.0709 (5)	
H50	1.2628	−0.2649	1.0921	0.085*	
C51	1.4023 (3)	−0.22336 (17)	1.14669 (14)	0.0836 (6)	
H51	1.4475	−0.2810	1.1902	0.100*	
C52	1.4454 (3)	−0.14910 (19)	1.14138 (15)	0.0945 (7)	
H52	1.5192	−0.1558	1.1814	0.113*	
C53	1.3792 (2)	−0.06407 (17)	1.07647 (14)	0.0808 (6)	
H53	1.4099	−0.0139	1.0735	0.097*	
C54	1.26825 (19)	−0.05043 (13)	1.01523 (11)	0.0557 (4)	
C55	1.2065 (2)	0.04182 (13)	0.94696 (12)	0.0569 (4)	
H55	1.2542	0.0861	0.9449	0.068*	
C56	1.09217 (19)	0.07140 (12)	0.88751 (11)	0.0534 (4)	
H56	1.0363	0.0303	0.8898	0.064*	
C57	1.0495 (2)	0.16541 (12)	0.81842 (12)	0.0554 (4)	
O1	0.4695 (2)	0.54064 (15)	0.16521 (13)	0.1011 (9)	0.797 (4)
O1'	0.4746 (8)	0.4998 (7)	0.3293 (9)	0.100 (3)	0.204 (4)
O2	0.01235 (14)	0.56378 (11)	0.14559 (9)	0.0789 (4)	
O3	0.05022 (15)	0.44048 (11)	0.36520 (9)	0.0831 (4)	
O4	0.84438 (19)	−0.17918 (15)	0.66161 (14)	0.0896 (7)	0.907 (4)
O4'	0.8606 (10)	−0.036 (2)	0.519 (2)	0.121 (9)	0.095 (4)
O5	0.50151 (15)	−0.03546 (9)	0.73550 (8)	0.0712 (4)	
O6	0.46332 (14)	0.12368 (9)	0.52412 (8)	0.0669 (4)	
O7	1.10487 (18)	0.22515 (9)	0.81922 (10)	0.0826 (4)	
O8	0.96687 (14)	0.04133 (8)	0.76207 (8)	0.0635 (3)	
O9	1.11850 (16)	−0.11450 (8)	0.96222 (8)	0.0692 (4)	
H17	0.4431 (7)	0.4810 (13)	0.327 (3)	0.083*	0.797 (4)
H19	0.4298 (5)	0.5422 (5)	0.1523 (6)	0.083*	0.204 (4)
H37	0.8275 (6)	−0.024 (3)	0.522 (3)	0.083*	0.907 (4)
H20	0.815 (2)	−0.166 (11)	0.662 (11)	0.083*	0.095 (4)

### Atomic displacement parameters (Å^2^)

**Table:**

	*U*^11^	*U*^22^	*U*^33^	*U*^12^	*U*^13^	*U*^23^
C1	0.0621 (12)	0.0419 (10)	0.0679 (12)	−0.0111 (8)	0.0108 (9)	−0.0222 (9)
C2	0.0787 (14)	0.0573 (12)	0.0780 (14)	−0.0147 (10)	0.0209 (12)	−0.0257 (11)
C3	0.108 (2)	0.0759 (15)	0.0725 (15)	−0.0216 (13)	0.0268 (14)	−0.0332 (12)
C4	0.114 (2)	0.0695 (14)	0.0671 (14)	−0.0258 (13)	0.0062 (13)	−0.0332 (11)
C5	0.0821 (14)	0.0606 (12)	0.0723 (13)	−0.0221 (10)	0.0007 (11)	−0.0308 (11)
C6	0.0680 (13)	0.0512 (11)	0.0638 (12)	−0.0162 (9)	0.0098 (10)	−0.0271 (9)
C7	0.0511 (11)	0.0862 (15)	0.0838 (14)	−0.0082 (10)	−0.0030 (10)	−0.0397 (12)
C8	0.0482 (11)	0.0897 (16)	0.0924 (15)	−0.0108 (10)	0.0067 (10)	−0.0482 (13)
C9	0.0614 (13)	0.0990 (18)	0.0937 (16)	−0.0342 (12)	0.0097 (11)	−0.0503 (14)
C10	0.0625 (13)	0.0915 (16)	0.0852 (15)	−0.0322 (11)	0.0170 (11)	−0.0378 (13)
C11	0.0589 (12)	0.0605 (12)	0.0649 (12)	−0.0069 (9)	0.0030 (9)	−0.0277 (10)
C12	0.0671 (13)	0.0722 (14)	0.0730 (13)	−0.0095 (10)	0.0118 (11)	−0.0352 (11)
C13	0.0893 (17)	0.0758 (15)	0.0682 (13)	−0.0053 (12)	0.0098 (12)	−0.0394 (11)
C14	0.0887 (17)	0.0839 (16)	0.0762 (15)	−0.0056 (13)	−0.0079 (12)	−0.0434 (13)
C15	0.0661 (13)	0.0657 (13)	0.0821 (15)	−0.0076 (10)	−0.0037 (11)	−0.0349 (11)
C16	0.0546 (11)	0.0507 (11)	0.0690 (12)	−0.0038 (8)	−0.0004 (9)	−0.0257 (9)
C17	0.0504 (11)	0.0567 (12)	0.0773 (14)	−0.0054 (9)	0.0035 (11)	−0.0239 (10)
C18	0.0493 (11)	0.0829 (15)	0.0690 (13)	−0.0173 (10)	0.0036 (10)	−0.0227 (11)
C19	0.0566 (12)	0.0562 (12)	0.0761 (13)	−0.0092 (9)	0.0065 (10)	−0.0239 (10)
C20	0.0573 (12)	0.0747 (14)	0.0786 (13)	−0.0071 (10)	−0.0052 (10)	−0.0521 (12)
C21	0.0588 (11)	0.0622 (12)	0.0732 (12)	−0.0139 (9)	−0.0088 (9)	−0.0358 (10)
C22	0.0760 (14)	0.0733 (15)	0.0978 (17)	−0.0102 (12)	−0.0179 (13)	−0.0440 (14)
C23	0.0973 (19)	0.0671 (15)	0.0985 (19)	−0.0282 (14)	−0.0157 (15)	−0.0203 (14)
C24	0.0984 (19)	0.0875 (19)	0.0934 (18)	−0.0382 (15)	0.0097 (15)	−0.0268 (16)
C25	0.0810 (15)	0.0785 (16)	0.0796 (15)	−0.0282 (12)	0.0141 (12)	−0.0299 (13)
C26	0.0610 (12)	0.0670 (13)	0.0699 (12)	−0.0207 (10)	0.0024 (10)	−0.0328 (11)
C27	0.0714 (13)	0.0829 (15)	0.0701 (12)	−0.0177 (11)	0.0212 (10)	−0.0469 (12)
C28	0.0715 (13)	0.0739 (14)	0.0701 (12)	−0.0128 (10)	0.0194 (10)	−0.0466 (11)
C29	0.0526 (11)	0.0845 (14)	0.0787 (13)	−0.0106 (10)	0.0152 (10)	−0.0457 (12)
C30	0.0570 (11)	0.0759 (13)	0.0704 (12)	−0.0012 (10)	0.0084 (9)	−0.0426 (11)
C31	0.0556 (11)	0.0716 (13)	0.0587 (11)	−0.0166 (9)	0.0090 (8)	−0.0433 (10)
C32	0.0712 (13)	0.0709 (14)	0.0661 (12)	−0.0156 (10)	0.0090 (10)	−0.0375 (11)
C33	0.0911 (17)	0.0847 (16)	0.0788 (15)	−0.0267 (14)	0.0202 (13)	−0.0353 (13)
C34	0.100 (2)	0.105 (2)	0.0972 (19)	−0.0329 (17)	0.0438 (15)	−0.0366 (17)
C35	0.0691 (14)	0.1033 (19)	0.0983 (17)	−0.0151 (13)	0.0281 (13)	−0.0532 (16)
C36	0.0560 (11)	0.0763 (14)	0.0653 (12)	−0.0148 (10)	0.0089 (9)	−0.0450 (11)
C37	0.0502 (11)	0.0862 (15)	0.0769 (13)	−0.0054 (11)	0.0042 (11)	−0.0561 (12)
C38	0.0540 (11)	0.0717 (13)	0.0677 (12)	−0.0020 (10)	0.0015 (9)	−0.0445 (11)
C39	0.0499 (10)	0.0532 (11)	0.0595 (10)	−0.0126 (8)	0.0129 (8)	−0.0282 (9)
C40	0.0667 (12)	0.0571 (12)	0.0801 (13)	−0.0163 (10)	0.0156 (11)	−0.0317 (11)
C41	0.0701 (14)	0.0611 (13)	0.0848 (15)	−0.0014 (11)	−0.0053 (12)	−0.0192 (12)
C42	0.0704 (14)	0.0776 (16)	0.0792 (14)	−0.0040 (12)	−0.0134 (11)	−0.0272 (13)
C43	0.0657 (12)	0.0718 (14)	0.0712 (13)	−0.0082 (10)	−0.0069 (10)	−0.0351 (11)
C44	0.0491 (10)	0.0569 (11)	0.0592 (11)	−0.0095 (8)	0.0053 (8)	−0.0290 (9)
C45	0.0749 (13)	0.0705 (13)	0.0748 (13)	−0.0138 (10)	−0.0067 (10)	−0.0474 (11)
C46	0.0723 (13)	0.0632 (12)	0.0829 (14)	−0.0085 (10)	−0.0070 (10)	−0.0501 (11)
C47	0.0722 (13)	0.0606 (12)	0.0876 (14)	−0.0209 (10)	0.0005 (11)	−0.0406 (11)
C48	0.0858 (14)	0.0536 (12)	0.0744 (13)	−0.0209 (10)	−0.0016 (11)	−0.0321 (10)
C49	0.0562 (11)	0.0631 (12)	0.0539 (10)	−0.0095 (9)	0.0047 (8)	−0.0321 (9)
C50	0.0760 (14)	0.0668 (13)	0.0653 (12)	−0.0101 (11)	0.0009 (11)	−0.0289 (11)
C51	0.0762 (15)	0.0821 (16)	0.0709 (14)	−0.0058 (12)	−0.0059 (11)	−0.0218 (12)
C52	0.0787 (16)	0.112 (2)	0.0822 (16)	−0.0230 (15)	−0.0214 (13)	−0.0337 (16)
C53	0.0742 (14)	0.0912 (17)	0.0804 (14)	−0.0287 (12)	−0.0087 (12)	−0.0376 (13)
C54	0.0499 (10)	0.0691 (12)	0.0569 (10)	−0.0167 (9)	0.0062 (8)	−0.0352 (10)
C55	0.0574 (11)	0.0632 (12)	0.0663 (11)	−0.0226 (9)	0.0090 (9)	−0.0399 (10)
C56	0.0574 (11)	0.0568 (11)	0.0588 (10)	−0.0195 (8)	0.0089 (9)	−0.0349 (9)
C57	0.0579 (11)	0.0554 (11)	0.0649 (11)	−0.0177 (9)	0.0146 (9)	−0.0362 (10)
O1	0.0545 (12)	0.139 (2)	0.0884 (14)	−0.0309 (12)	0.0096 (10)	−0.0315 (13)
O1'	0.079 (6)	0.142 (8)	0.090 (6)	−0.063 (5)	0.018 (4)	−0.047 (6)
O2	0.0511 (8)	0.1105 (12)	0.0688 (9)	−0.0109 (7)	0.0041 (6)	−0.0391 (8)
O3	0.0572 (8)	0.1169 (12)	0.0693 (9)	−0.0312 (8)	0.0090 (7)	−0.0337 (8)
O4	0.0751 (12)	0.0944 (13)	0.0961 (13)	0.0129 (9)	0.0005 (9)	−0.0541 (10)
O4'	0.052 (10)	0.097 (15)	0.162 (19)	0.027 (10)	0.005 (12)	−0.034 (13)
O5	0.0779 (9)	0.0655 (9)	0.0703 (8)	−0.0123 (7)	0.0215 (7)	−0.0338 (7)
O6	0.0605 (8)	0.0707 (9)	0.0639 (8)	−0.0012 (6)	0.0161 (6)	−0.0324 (7)
O7	0.1009 (11)	0.0641 (9)	0.0927 (10)	−0.0327 (8)	−0.0031 (8)	−0.0382 (8)
O8	0.0721 (8)	0.0557 (8)	0.0694 (8)	−0.0076 (6)	−0.0115 (6)	−0.0363 (7)
O9	0.0904 (10)	0.0537 (8)	0.0676 (8)	−0.0195 (7)	−0.0140 (7)	−0.0289 (6)

### Geometric parameters (Å, º)

**Table:**

C1—C2	1.388 (3)	C29—H29A	0.9700
C1—C6	1.406 (3)	C29—H29B	0.9700
C1—C19	1.487 (3)	C30—O6	1.428 (2)
C2—C3	1.372 (3)	C30—H30A	0.9700
C2—H2	0.9300	C30—H30B	0.9700
C3—C4	1.371 (3)	C31—O6	1.358 (2)
C3—H3	0.9300	C31—C32	1.381 (3)
C4—C5	1.370 (3)	C31—C36	1.402 (2)
C4—H4	0.9300	C32—C33	1.372 (3)
C5—C6	1.386 (3)	C32—H32	0.9300
C5—H5	0.9300	C33—C34	1.364 (3)
C6—O2	1.353 (2)	C33—H33	0.9300
C7—O2	1.433 (2)	C34—C35	1.379 (3)
C7—C8	1.499 (3)	C34—H34	0.9300
C7—H7A	0.9700	C35—C36	1.394 (3)
C7—H7B	0.9700	C35—H35	0.9300
C8—C9	1.521 (3)	C36—C37	1.464 (3)
C8—H8A	0.9700	C37—O4'	1.276 (9)
C8—H8B	0.9700	C37—C38	1.336 (3)
C9—C10	1.500 (3)	C37—H37	0.935 (5)
C9—H9A	0.9700	C38—H38	0.9300
C9—H9B	0.9700	C39—C40	1.399 (3)
C10—O3	1.425 (2)	C39—C44	1.407 (2)
C10—H10A	0.9700	C39—C57	1.496 (2)
C10—H10B	0.9700	C40—C41	1.375 (3)
C11—O3	1.355 (2)	C40—H40	0.9300
C11—C12	1.388 (3)	C41—C42	1.366 (3)
C11—C16	1.404 (3)	C41—H41	0.9300
C12—C13	1.369 (3)	C42—C43	1.364 (3)
C12—H12	0.9300	C42—H42	0.9300
C13—C14	1.370 (3)	C43—C44	1.391 (3)
C13—H13	0.9300	C43—H43	0.9300
C14—C15	1.376 (3)	C44—O8	1.358 (2)
C14—H14	0.9300	C45—O8	1.440 (2)
C15—C16	1.391 (3)	C45—C46	1.497 (3)
C15—H15	0.9300	C45—H45A	0.9700
C16—C17	1.468 (3)	C45—H45B	0.9700
C17—O1'	1.331 (6)	C46—C47	1.524 (3)
C17—C18	1.337 (3)	C46—H46A	0.9700
C17—H17	0.933 (5)	C46—H46B	0.9700
C18—C19	1.425 (3)	C47—C48	1.501 (3)
C18—H18	0.9300	C47—H47A	0.9700
C19—O1	1.258 (2)	C47—H47B	0.9700
C19—H19	0.930 (5)	C48—O9	1.428 (2)
C20—O4	1.235 (2)	C48—H48A	0.9700
C20—C38	1.451 (3)	C48—H48B	0.9700
C20—C21	1.497 (3)	C49—O9	1.355 (2)
C20—H20	0.930 (5)	C49—C50	1.379 (3)
C21—C22	1.397 (3)	C49—C54	1.406 (2)
C21—C26	1.400 (3)	C50—C51	1.377 (3)
C22—C23	1.380 (3)	C50—H50	0.9300
C22—H22	0.9300	C51—C52	1.359 (3)
C23—C24	1.364 (4)	C51—H51	0.9300
C23—H23	0.9300	C52—C53	1.375 (3)
C24—C25	1.361 (3)	C52—H52	0.9300
C24—H24	0.9300	C53—C54	1.388 (3)
C25—C26	1.395 (3)	C53—H53	0.9300
C25—H25	0.9300	C54—C55	1.460 (3)
C26—O5	1.357 (2)	C55—C56	1.325 (2)
C27—O5	1.433 (2)	C55—H55	0.9300
C27—C28	1.496 (3)	C56—C57	1.462 (2)
C27—H27A	0.9700	C56—H56	0.9300
C27—H27B	0.9700	C57—O7	1.228 (2)
C28—C29	1.527 (3)	O1—H19	0.420 (5)
C28—H28A	0.9700	O1'—H17	0.484 (15)
C28—H28B	0.9700	O4—H20	0.31 (3)
C29—C30	1.500 (3)	O4'—H37	0.342 (12)
			
C2—C1—C6	117.19 (18)	C28—C29—H29B	108.8
C2—C1—C19	116.88 (18)	H29A—C29—H29B	107.7
C6—C1—C19	125.91 (17)	O6—C30—C29	105.39 (16)
C3—C2—C1	122.5 (2)	O6—C30—H30A	110.7
C3—C2—H2	118.7	C29—C30—H30A	110.7
C1—C2—H2	118.7	O6—C30—H30B	110.7
C4—C3—C2	119.1 (2)	C29—C30—H30B	110.7
C4—C3—H3	120.4	H30A—C30—H30B	108.8
C2—C3—H3	120.4	O6—C31—C32	123.27 (17)
C5—C4—C3	120.5 (2)	O6—C31—C36	115.58 (17)
C5—C4—H4	119.7	C32—C31—C36	121.15 (17)
C3—C4—H4	119.7	C33—C32—C31	120.1 (2)
C4—C5—C6	120.5 (2)	C33—C32—H32	119.9
C4—C5—H5	119.8	C31—C32—H32	119.9
C6—C5—H5	119.8	C34—C33—C32	120.4 (2)
O2—C6—C5	122.91 (18)	C34—C33—H33	119.8
O2—C6—C1	116.99 (17)	C32—C33—H33	119.8
C5—C6—C1	120.09 (18)	C33—C34—C35	119.5 (2)
O2—C7—C8	105.59 (16)	C33—C34—H34	120.2
O2—C7—H7A	110.6	C35—C34—H34	120.2
C8—C7—H7A	110.6	C34—C35—C36	122.2 (2)
O2—C7—H7B	110.6	C34—C35—H35	118.9
C8—C7—H7B	110.6	C36—C35—H35	118.9
H7A—C7—H7B	108.8	C35—C36—C31	116.46 (19)
C7—C8—C9	113.87 (18)	C35—C36—C37	118.94 (18)
C7—C8—H8A	108.8	C31—C36—C37	124.58 (17)
C9—C8—H8A	108.8	O4'—C37—C38	119.5 (18)
C7—C8—H8B	108.8	O4'—C37—C36	110.8 (18)
C9—C8—H8B	108.8	C38—C37—C36	128.95 (17)
H8A—C8—H8B	107.7	O4'—C37—H37	1 (5)
C10—C9—C8	114.65 (18)	C38—C37—H37	121 (3)
C10—C9—H9A	108.6	C36—C37—H37	109 (3)
C8—C9—H9A	108.6	C37—C38—C20	123.12 (18)
C10—C9—H9B	108.6	C37—C38—H38	118.4
C8—C9—H9B	108.6	C20—C38—H38	118.4
H9A—C9—H9B	107.6	C40—C39—C44	116.89 (17)
O3—C10—C9	105.56 (16)	C40—C39—C57	116.85 (16)
O3—C10—H10A	110.6	C44—C39—C57	126.25 (16)
C9—C10—H10A	110.6	C41—C40—C39	122.10 (19)
O3—C10—H10B	110.6	C41—C40—H40	119.0
C9—C10—H10B	110.6	C39—C40—H40	119.0
H10A—C10—H10B	108.8	C42—C41—C40	119.6 (2)
O3—C11—C12	123.02 (18)	C42—C41—H41	120.2
O3—C11—C16	116.58 (17)	C40—C41—H41	120.2
C12—C11—C16	120.39 (19)	C43—C42—C41	120.6 (2)
C13—C12—C11	120.5 (2)	C43—C42—H42	119.7
C13—C12—H12	119.7	C41—C42—H42	119.7
C11—C12—H12	119.7	C42—C43—C44	120.7 (2)
C12—C13—C14	120.4 (2)	C42—C43—H43	119.7
C12—C13—H13	119.8	C44—C43—H43	119.7
C14—C13—H13	119.8	O8—C44—C43	122.17 (16)
C13—C14—C15	119.3 (2)	O8—C44—C39	117.63 (15)
C13—C14—H14	120.4	C43—C44—C39	120.16 (17)
C15—C14—H14	120.4	O8—C45—C46	107.12 (14)
C14—C15—C16	122.5 (2)	O8—C45—H45A	110.3
C14—C15—H15	118.7	C46—C45—H45A	110.3
C16—C15—H15	118.7	O8—C45—H45B	110.3
C15—C16—C11	116.87 (18)	C46—C45—H45B	110.3
C15—C16—C17	118.42 (18)	H45A—C45—H45B	108.5
C11—C16—C17	124.71 (18)	C45—C46—C47	114.28 (16)
O1'—C17—C18	121.0 (6)	C45—C46—H46A	108.7
O1'—C17—C16	111.1 (6)	C47—C46—H46A	108.7
C18—C17—C16	127.94 (18)	C45—C46—H46B	108.7
O1'—C17—H17	14.2 (12)	C47—C46—H46B	108.7
C18—C17—H17	116 (3)	H46A—C46—H46B	107.6
C16—C17—H17	114 (3)	C48—C47—C46	113.72 (17)
C17—C18—C19	124.49 (19)	C48—C47—H47A	108.8
C17—C18—H18	117.8	C46—C47—H47A	108.8
C19—C18—H18	117.8	C48—C47—H47B	108.8
O1—C19—C18	120.5 (2)	C46—C47—H47B	108.8
O1—C19—C1	116.98 (19)	H47A—C47—H47B	107.7
C18—C19—C1	122.37 (18)	O9—C48—C47	105.23 (15)
O1—C19—H19	13.9 (6)	O9—C48—H48A	110.7
C18—C19—H19	134.5 (6)	C47—C48—H48A	110.7
C1—C19—H19	103.1 (6)	O9—C48—H48B	110.7
O4—C20—C38	121.5 (2)	C47—C48—H48B	110.7
O4—C20—C21	117.6 (2)	H48A—C48—H48B	108.8
C38—C20—C21	120.96 (17)	O9—C49—C50	123.04 (17)
O4—C20—H20	2 (10)	O9—C49—C54	115.83 (16)
C38—C20—H20	120 (10)	C50—C49—C54	121.12 (17)
C21—C20—H20	119 (10)	C51—C50—C49	120.0 (2)
C22—C21—C26	116.9 (2)	C51—C50—H50	120.0
C22—C21—C20	116.97 (19)	C49—C50—H50	120.0
C26—C21—C20	126.07 (18)	C52—C51—C50	120.4 (2)
C23—C22—C21	122.0 (2)	C52—C51—H51	119.8
C23—C22—H22	119.0	C50—C51—H51	119.8
C21—C22—H22	119.0	C51—C52—C53	119.7 (2)
C24—C23—C22	119.6 (2)	C51—C52—H52	120.2
C24—C23—H23	120.2	C53—C52—H52	120.2
C22—C23—H23	120.2	C52—C53—C54	122.4 (2)
C25—C24—C23	120.6 (2)	C52—C53—H53	118.8
C25—C24—H24	119.7	C54—C53—H53	118.8
C23—C24—H24	119.7	C53—C54—C49	116.41 (18)
C24—C25—C26	120.5 (2)	C53—C54—C55	119.04 (17)
C24—C25—H25	119.8	C49—C54—C55	124.54 (16)
C26—C25—H25	119.8	C56—C55—C54	129.21 (17)
O5—C26—C25	122.03 (19)	C56—C55—H55	115.4
O5—C26—C21	117.57 (17)	C54—C55—H55	115.4
C25—C26—C21	120.4 (2)	C55—C56—C57	123.24 (16)
O5—C27—C28	106.78 (15)	C55—C56—H56	118.4
O5—C27—H27A	110.4	C57—C56—H56	118.4
C28—C27—H27A	110.4	O7—C57—C56	121.13 (17)
O5—C27—H27B	110.4	O7—C57—C39	118.62 (16)
C28—C27—H27B	110.4	C56—C57—C39	120.25 (15)
H27A—C27—H27B	108.6	C19—O1—H19	32.3 (13)
C27—C28—C29	114.89 (17)	C17—O1'—H17	28.3 (18)
C27—C28—H28A	108.5	C6—O2—C7	121.20 (15)
C29—C28—H28A	108.5	C11—O3—C10	121.56 (16)
C27—C28—H28B	108.5	C20—O4—H20	5 (10)
C29—C28—H28B	108.5	C37—O4'—H37	4 (10)
H28A—C28—H28B	107.5	C26—O5—C27	119.54 (15)
C30—C29—C28	113.87 (17)	C31—O6—C30	120.30 (15)
C30—C29—H29A	108.8	C44—O8—C45	119.07 (14)
C28—C29—H29A	108.8	C49—O9—C48	120.23 (14)
C30—C29—H29B	108.8		
			
C6—C1—C2—C3	−0.6 (3)	C32—C31—C36—C35	2.4 (3)
C19—C1—C2—C3	−179.13 (19)	O6—C31—C36—C37	1.0 (3)
C1—C2—C3—C4	1.2 (3)	C32—C31—C36—C37	−178.98 (17)
C2—C3—C4—C5	−1.1 (3)	C35—C36—C37—O4'	−5.5 (3)
C3—C4—C5—C6	0.4 (3)	C31—C36—C37—O4'	175.9 (3)
C4—C5—C6—O2	179.27 (18)	C35—C36—C37—C38	−175.71 (19)
C4—C5—C6—C1	0.2 (3)	C31—C36—C37—C38	5.7 (3)
C2—C1—C6—O2	−179.23 (16)	O4'—C37—C38—C20	14.1 (4)
C19—C1—C6—O2	−0.9 (3)	C36—C37—C38—C20	−176.43 (17)
C2—C1—C6—C5	−0.1 (3)	O4—C20—C38—C37	−14.3 (3)
C19—C1—C6—C5	178.27 (17)	C21—C20—C38—C37	163.96 (17)
O2—C7—C8—C9	−69.8 (2)	C44—C39—C40—C41	−0.1 (3)
C7—C8—C9—C10	135.73 (19)	C57—C39—C40—C41	178.80 (17)
C8—C9—C10—O3	−66.2 (2)	C39—C40—C41—C42	−1.0 (3)
O3—C11—C12—C13	179.56 (19)	C40—C41—C42—C43	1.1 (3)
C16—C11—C12—C13	−0.2 (3)	C41—C42—C43—C44	0.0 (3)
C11—C12—C13—C14	1.1 (3)	C42—C43—C44—O8	−178.90 (18)
C12—C13—C14—C15	−0.9 (3)	C42—C43—C44—C39	−1.2 (3)
C13—C14—C15—C16	−0.1 (3)	C40—C39—C44—O8	179.03 (15)
C14—C15—C16—C11	0.9 (3)	C57—C39—C44—O8	0.2 (3)
C14—C15—C16—C17	−178.48 (18)	C40—C39—C44—C43	1.2 (2)
O3—C11—C16—C15	179.47 (17)	C57—C39—C44—C43	−177.58 (17)
C12—C11—C16—C15	−0.7 (3)	O8—C45—C46—C47	71.1 (2)
O3—C11—C16—C17	−1.2 (3)	C45—C46—C47—C48	−133.37 (17)
C12—C11—C16—C17	178.60 (18)	C46—C47—C48—O9	68.7 (2)
C15—C16—C17—O1'	−6.6 (5)	O9—C49—C50—C51	178.75 (18)
C11—C16—C17—O1'	174.1 (5)	C54—C49—C50—C51	0.2 (3)
C15—C16—C17—C18	173.84 (18)	C49—C50—C51—C52	0.3 (3)
C11—C16—C17—C18	−5.5 (3)	C50—C51—C52—C53	−0.5 (4)
O1'—C17—C18—C19	2.8 (5)	C51—C52—C53—C54	0.2 (4)
C16—C17—C18—C19	−177.69 (17)	C52—C53—C54—C49	0.3 (3)
C17—C18—C19—O1	5.5 (2)	C52—C53—C54—C55	−178.4 (2)
C17—C18—C19—C1	−170.82 (18)	O9—C49—C54—C53	−179.17 (16)
C2—C1—C19—O1	17.7 (3)	C50—C49—C54—C53	−0.5 (3)
C6—C1—C19—O1	−160.68 (19)	O9—C49—C54—C55	−0.5 (2)
C2—C1—C19—C18	−165.91 (16)	C50—C49—C54—C55	178.11 (17)
C6—C1—C19—C18	15.7 (3)	C53—C54—C55—C56	−173.20 (19)
O4—C20—C21—C22	−18.2 (3)	C49—C54—C55—C56	8.2 (3)
C38—C20—C21—C22	163.50 (17)	C54—C55—C56—C57	−175.25 (16)
O4—C20—C21—C26	159.25 (18)	C55—C56—C57—O7	−10.3 (3)
C38—C20—C21—C26	−19.0 (3)	C55—C56—C57—C39	169.22 (16)
C26—C21—C22—C23	−0.7 (3)	C40—C39—C57—O7	−19.8 (2)
C20—C21—C22—C23	177.04 (18)	C44—C39—C57—O7	159.01 (17)
C21—C22—C23—C24	−1.7 (3)	C40—C39—C57—C56	160.68 (15)
C22—C23—C24—C25	2.4 (4)	C44—C39—C57—C56	−20.5 (3)
C23—C24—C25—C26	−0.7 (4)	C5—C6—O2—C7	−5.4 (3)
C24—C25—C26—O5	−179.8 (2)	C1—C6—O2—C7	173.73 (17)
C24—C25—C26—C21	−1.7 (3)	C8—C7—O2—C6	175.49 (17)
C22—C21—C26—O5	−179.53 (17)	C12—C11—O3—C10	−0.2 (3)
C20—C21—C26—O5	3.0 (3)	C16—C11—O3—C10	179.64 (18)
C22—C21—C26—C25	2.3 (3)	C9—C10—O3—C11	176.79 (18)
C20—C21—C26—C25	−175.11 (18)	C25—C26—O5—C27	−6.7 (3)
O5—C27—C28—C29	72.9 (2)	C21—C26—O5—C27	175.18 (16)
C27—C28—C29—C30	−133.58 (18)	C28—C27—O5—C26	−173.76 (16)
C28—C29—C30—O6	68.8 (2)	C32—C31—O6—C30	0.3 (3)
O6—C31—C32—C33	178.75 (17)	C36—C31—O6—C30	−179.69 (16)
C36—C31—C32—C33	−1.3 (3)	C29—C30—O6—C31	−176.31 (15)
C31—C32—C33—C34	−0.5 (3)	C43—C44—O8—C45	−4.8 (3)
C32—C33—C34—C35	1.1 (4)	C39—C44—O8—C45	177.49 (16)
C33—C34—C35—C36	0.1 (4)	C46—C45—O8—C44	−171.89 (15)
C34—C35—C36—C31	−1.8 (3)	C50—C49—O9—C48	8.7 (3)
C34—C35—C36—C37	179.5 (2)	C54—C49—O9—C48	−172.64 (16)
O6—C31—C36—C35	−177.69 (17)	C47—C48—O9—C49	177.11 (15)

### Hydrogen-bond geometry (Å, º)

**Table:**

*D*—H···*A*	*D*—H	H···*A*	*D*···*A*	*D*—H···*A*
C2—H2···O1	0.93	2.37	2.712 (3)	101
C18—H18···O2	0.93	2.15	2.746 (2)	121
C18—H18···O3	0.93	2.12	2.763 (2)	126
C38—H38···O5	0.93	2.24	2.753 (2)	114
C38—H38···O6	0.93	2.13	2.753 (3)	123
C55—H55···O7	0.93	2.50	2.829 (2)	101
C56—H56···O8	0.93	2.23	2.766 (2)	116
C56—H56···O9	0.93	2.15	2.764 (2)	122
